# Brain Tumor Detection and Classification on MR Images by a Deep Wavelet Auto-Encoder Model

**DOI:** 10.3390/diagnostics11091589

**Published:** 2021-08-31

**Authors:** Isselmou Abd El Kader, Guizhi Xu, Zhang Shuai, Sani Saminu, Imran Javaid, Isah Salim Ahmad, Souha Kamhi

**Affiliations:** State Key Laboratory of Reliability and Intelligence of Electrical Equipment, Hebei University of Technology, Tianjin 300130, China; zs@hebut.edu.cn (Z.S.); sansam4k@gmail.com (S.S.); imranpolitely111@gmail.com (I.J.); isahsalimahmad@gmail.com (I.S.A.); souhakamhi@outlook.com (S.K.)

**Keywords:** MRI, brain tumor, detection, classification, seed growing, segmentation, deep wavelet auto-encoder

## Abstract

The process of diagnosing brain tumors is very complicated for many reasons, including the brain’s synaptic structure, size, and shape. Machine learning techniques are employed to help doctors to detect brain tumor and support their decisions. In recent years, deep learning techniques have made a great achievement in medical image analysis. This paper proposed a deep wavelet autoencoder model named “DWAE model”, employed to divide input data slice as a tumor (abnormal) or no tumor (normal). This article used a high pass filter to show the heterogeneity of the MRI images and their integration with the input images. A high median filter was utilized to merge slices. We improved the output slices’ quality through highlight edges and smoothened input MR brain images. Then, we applied the seed growing method based on 4-connected since the thresholding cluster equal pixels with input MR data. The segmented MR image slices provide two two-layer using the proposed deep wavelet auto-encoder model. We then used 200 hidden units in the first layer and 400 hidden units in the second layer. The softmax layer testing and training are performed for the identification of the MR image normal and abnormal. The contribution of the deep wavelet auto-encoder model is in the analysis of pixel pattern of MR brain image and the ability to detect and classify the tumor with high accuracy, short time, and low loss validation. To train and test the overall performance of the proposed model, we utilized 2500 MR brain images from BRATS2012, BRATS2013, BRATS2014, BRATS2015, 2015 challenge, and ISLES, which consists of normal and abnormal images. The experiments results show that the proposed model achieved an accuracy of 99.3%, loss validation of 0.1, low FPR and FNR values. This result demonstrates that the proposed DWAE model can facilitate the automatic detection of brain tumors.

## 1. Introduction

The brain tumor and its analysis are of extraordinary interest because of the developing innovation in medical image processing. As indicated by the overview led by the National Brain Tumor Foundation (NBTF), the improvement of brain tumor diagnosis among patients and the death rate due to brain tumors is succeeding earlier year’s insights across the globe [1,2]. The latest advances in machine learning (especially deep learning) help identify, classify, and measure patterns in medical images. The core of these developments is by using hierarchical feature representations learned only from data rather than manually designed features based on specific domain knowledge. Likewise, a few works have proposed frameworks or models to feature the brain tumor zone in recent years, which might be trailed by stages such as outcome predictions, classification, and treatment planning. Brain tumor segmentation in medical image processing is necessary and generally governed by factors such as missing boundaries, noise, and low contrast. MRI segmentation using learning strategies and pattern recognition technology is very successful in analyzing brain images. Technically speaking, the method is a parametric model that considers the functions selected based on the density function [3]. Early screening of such brain tumors issues is crucial to encourage convenient therapy and sound living with contemporary clinical imaging modalities. The most common modalities that are utilized to analyze the tumor in the brain are positron emission tomography (PET), magnetic resonance imaging (MRI), and computed tomography (CT) [2].

Magnetic Resonance Imaging (MRI) is a well-known medical device used to diagnose and analyze many diseases such as brain tumors, neurological diseases, epilepsy, etc. Usually, a system completely processed by hardware/computer helps automate this process to obtain accurate and fast results. On the other hand, image segmentation is the main task of various computer vision and image processing implementations. The hypothesis of the hash process divides the image into different areas according to some measures for further processing [4,5]. Detection of brain abnormalities is usually done manually using MRI imaging by medical experts. The large-scale manual examination method can often lead to misinterpretation due to some factors such as fatigue and excessive abundance of MRI slices. In addition, it is non-repeatable and results in intra- and inter-reader variability. Alleviating these concerns requires developing a detection system method to diagnose various brain abnormalities. It also helps in promoting fast, reliable, and accurate analysis and supports the clinicians in their final selection process. Machine learning techniques are mainly used to design and automate systems that have enjoyed spectacular success in recent decades. Many methods (also known as automatic detection of pathological brain systems) have been formulated to classify the brain’s different MRI scans. These diagrams mainly focus on solving two brain types based on MRI classification disorders, namely binary and multiclass. In the binary category, brain MRI scans are classified as either pathological (abnormal) or normal.

## 2. Related Work

El-Dahshan et al. [6] proposed a method using two-dimensional deep wavelet transform (2D-DWT) and principal component analysis (PCA) to extract salient features. They employed a feed-forward neural network (FNN) and k-nearest neighbor (KNN) individually for classification. Das et al. [7] developed a model based on ripplet transform (RT), and features are recharging to the least squares SVM (LS-SVM) classifier. A fluid vector is utilized for tumor detection by using T1 weighted images [8]. Diffusion coefficients have been used to identify the tumor based on the diffusion tensor images [9]. Researchers have done a lot of work to extract and reduce an optimal feature of brain tumors; however, removing and selecting the optimum feature remains a complicated task because the number of features increases the association. In addition, selecting the training and testing samples is also a challenge in obtaining good results [10,11]. Amin et al. [12] proposed a distinctive method for MR brain detection and classification. First, the Gaussian filter was used to eliminate noise; then, brain image features are extracted by embedded, cyclic, contrast, block appearance, etc., for segmentation processing—cross-validation technology for classification. Chen et al. [13] suggested a hybrid between fuzzy clustering and Markov random field and integrated the original image’s fuzzy clustering membership into Markov random field function. This hybrid approach is the segmentation of the supporting information, and it gives good efficiency.

Chen et al. [14] proposed a wavelet-like Auto Encoder (WAE) based on a neural network, which analyzes the original image into a low-resolution image for classification. These low-resolution channels or images are further used to input the Convolutional Neural Network (CNN) to reduce computational complexity without altering the accuracy factor. There are several deep convolutional neural networks and fully convolutional neural networks (FCNN) [15], two pathway cascaded neural network model [16], auto-encoder [17], (CNN’s) [18,19], DeconvNets (EDD) [20], and three-dimensional convolutional neural network (CNN) [21] are utilized for MRI images analysis. Two tracks CNN model [18] is used for the prediction of brain tissues. Automatic multimodal brain tumor detection and classification are discussed [22]. Binary CNN is used for complete tumor area prediction [23]. The patch-based approach is used for medical image analysis [24]. The victories of the deep learning model were a game over lately, especially in 2012, Alexnet, the model that won the Imagenet competition, was an important deciding opinion. The most significant difference between a deep learning network and an artificial neural network is that a deep learning network consists of several layers [25]. In addition, the capsule network is vastly used [26], where the routing agreement process performs learning. The capsule network is excellent for classification images compared to the CNN model. The pooling in capsule networks is not performed for down sampling, as further improvement in tumor classification can also be utilized for the fusion of deep learning and handcrafted features [27].

Korfiatis et al. [28] proposed a deep convolutional neural network method based on autoencoders to segment increased signal areas in fluid-attenuated inversion recovery MRI images. They trained automatic convolutional encoders on the BRATS Benchmark dataset to segment the brain tumor image, and the accuracy was evaluated on a dataset with three retail experts. They used the simultaneous truth and performance level estimation (STAPLE) algorithm to provide the ground truth for comparison. The Jaccard coefficient, dice coefficient, true-positive fraction, and false-negative fraction values were calculated. The proposed method was within the inter-observer variability concerning true positive fraction, Dice, and Jaccard. The developed approach can be utilized to output automatic segmentation of the tumor area responsible for signal-increased fluid-attenuated inversion recovery areas.

Kumar et al. [29] proposed a compression technique based on a deep wavelet autoencoder, which combines the fundamental feature reduction property of the auto encoder and the image decomposition property of wavelet transform. These methods significantly affect the feature set’s size for undertaking another classification task using DNN. A brain image database was obtained, and the proposed DWA-DNN image classifier was considered. They compared the DWA-DNN classifier with the other classifiers such as DNN and autoencoder and achieved better results. Deep Nayaka et al. [30] proposed a deep neural network method where a stacked random vector functional link (RVFL) based autoencoder (SRVFL-AE) is used to identify the different class brain abnormalities. The autoencoders RVFL are the building blocks for their proposed SRVFL-AE. The main objective of selecting RVFL as a critical component of the SRVFL-AE is to improve learning speed and generalizability compared to deep learning methods based on the autoencoder. Moreover, they incorporated a ReLU (Rectified Linear Unit) activation function into the deep network, which they proposed for better-hidden representation of input features and better speed. To assess the effectiveness of their approach, they took two standard datasets of MD-1 and MD-2 MRI data. Their proposal approach achieved an accuracy of 96.67% and 95% on the MD-1 and MD-2 datasets.

Mishra et al. [31] proposed an efficient method for magnetic resonance imaging (MRI) brain image classification based on different wavelet transforms such as discrete wavelet transform (DWT) and stationary wavelet transforms (SWT). Dual-tree M-band wavelet transform (DMWT) was used for feature extraction and selection of coefficients for classification using support vector machine classifiers. They decomposed the normal and abnormal MRI brain image features through deep wavelet transform (DWT), SWT, and DWT. The results of their proposed method achieved an accuracy of 98% for MR brain images classification. Amin et al. [32] suggested a deep learning model to predict input slices as a tumor (unhealthy)/non-tumor (healthy). They used a high pass filter image to distinguish the MR slices’ in the homogeneities domain effect and integrated them with the input slices. Then, they applied a median filter to fuse the slices. They improved the quality of the resulting slices with smooth and highlighted edges of the input slices. They segmented the slices to the fine-tuned two layers proposed stacked sparse autoencoder (SSAE) approaches. They selected the hyperparametrs of the model after extensive experiments. In the first layer, they used 200 hidden units, and on the second layer, 400 hidden units. They tested the model on a softmax layer to predict from images with tumors and no tumors. They trained their model using BRATS datasets, i.e., 2012 (challenge and synthetic), 2013, and 2013 Leaderboard, 2014, and 2015 datasets.

Raja et al. [33] developed a brain tumor classification using a hybrid deep autoencoder with a Bayesian fuzzy clustering-based segmentation method. They carried out a pre-processing step with a non-local mean filter to reduce noise. They used the BFC (Bayesian fuzzy clustering) method for the segmentation of brain tumors. After segmentation, they used robust features such as information-theoretic measures, scattering transform (ST), and wavelet packet Tsallis entropy (WPT) approaches for the feature extraction process. Finally, they used a hybrid scheme of the DAE (deep autoencoder) based JOA (Jaya optimization algorithm) with a softmax regression technique to classify the tumor area for the brain tumor classification process. Their simulation result was conducted through the BRATS 2015 database.

Arunkumar et al. [34] proposed an improved automated brain tumor segmentation and detection method using the ANN model. They used MR data without human mediation by applying the best qualities for the preparatory detection of brain tumors. Their brain tumor segmentation technique consists of three noteworthy improvement focuses. Firstly, they used K-means clustering as part of the principal organization in MR data to distinguish the areas of the district in light of their grayscale. Secondly, they used ANN to select the correct object because of the training step. Thirdly, the tissue characteristics of the brain tumor area were removed to the mitotic stage. In recognition of brain tumors, gray-scale features analyze and diagnose brain tumors to distinguish benign and malignant tumors. Their model evaluates and compares with the SVM method segmentation outcomes and brain detection. Their model achieved an accuracy of 94.07%, sensitivity of 90.09%, and specificity of 96.78%.

Arunkumar et al. [35] suggested a novel segmentation study for brain tissues using MR images. Their methods consist of three computer vision fiction steps: enhancing images, segmenting images, and filtering out non-ROI based on the texture and HOG features. A fully automated MRI-based brain tumor segmentation and classification method is based on a model that uses artificial neural networks to locate an ROI accurately. Therefore, the non-ROI filtering process was used for histogram examination to avoid non-ROI and identify the correct object in brain MRI. However, histological features are used to determine the type of tumor. Two hundred MRI cases were used to compare the automatic and manual segmentation processes. The results show that fully automated trainable model-based segmentation is superior to manual methods and brain recognition using ROI texture features. Their model achieved a precision of 92.14%, with 89% sensitivity and 94% specificity.

Osama et al. [36] proposed a deep learning model that can predict mild cognitive impairment (MCI), early MCI (EMCI), late MCI (LMCI), and AD Alzheimer’s disease neuroimaging project. (ADNI) An fMRI data set consisting of 138 subjects was used for the assessment. Their fine-tuned ResNet18 network model achieved an accuracy of 99.99%, 99.95%, and 99.95% on EMCI vs. AD, LMCI vs. AD, and MCI vs. EMCI classification scenarios, respectively.

Huang et al. [37] proposed a differential feature map (DFM) block for brain tumor detection using MR images. The DFM blocks are combined with stress and excitation (SE) blocks to form a Differential Characteristic Neural Network (DFNN). First, they applied an automatic image correction method to make the symmetry axis of the MRI image of the brain approximately parallel to the vertical axis. In addition, DFNN was created to divide brain MRI images into two categories: “abnormal” and “normal”. Their experimental results show that the average accuracy of the proposed system on the two databases can reach 99.2% and 98%, and the introduction of the proposed DFM block can increase the average accuracy of the two databases by 1.8% and 1.3%, respectively.

Rundo et al. [38] suggested a fully automatic model for necrosis extraction (NeXt) based on the Fuzzy C-Means algorithm after the GTV segmentation. Unsupervised machine learning technology was used to detect and identify necrotic areas in heterogeneous cancers. Complete treatment pipeline is an integrated two-step segmentation method that can be used to support neuroradiology. They used NeXt for dose escalation, allowing more selective strategies to increase radiation dose in areas resistant to radiation and hypoxia. In addition, NeXt only analyzes contrast-enhanced T1 MRI images and does not require multispectral MRI data, which represents a clinically feasible solution. Their study considers an MR database that consists of 32 metastatic brain cancers, wherein 20 tumors present neuroses. The segmentation accuracy of the NeXt model was evaluated based on spatial overlap-based and distance-based metrics values and achieved a dice similarity coefficient of 95.93% ± 4.23% and mean absolute distance of 0.225 ± 0.229 (pixels).

Mekhmoukh et al. [39] proposed a novel segmentation approach based on Particle Swarm Optimization (PSO) and outlier rejection combined with the level set. The traditional algorithm for brain tumor segmentation of the MR database is the fuzzy c-means (FCM) algorithm. The membership function in this traditional algorithm is sensitive to external factors and does not incorporate spatial information into the image. The algorithm is very sensitive to noise and unevenness in the image, and it relies on the initialization of the centroid. To improve the external suppression of traditional FCM aggregation algorithms and reduce noise sensitivity, the authors presented a new extended FCM algorithm for image segmentation. In general, in the FCM algorithm, the initial cluster centers are selected randomly; with the help of the PSO algorithm, the centers of the clusters are selected optimally. Their algorithm also takes into consideration spatial neighborhood information. Their model achieved excellent effectiveness.

Han [40] proposed an unsupervised medical anomaly network model to detect unsupervised medical anomalies (MADGAN). A new two-step method that uses GAN-based multiple contiguous MRI slice reconstruction to detect brain abnormalities at different stages on multi-structured MRI: (reconstruction) Wasserstein loss with graded penalty + 100, $l_{1}loss-1$. Trained on three axial MRI slices of a healthy brain to rebuild 3-Reconstruct healthy/abnormal invisible scans; (diagnostics) average $l_{2}loss-2$ per scan characterizes them, by comparing baseline truth/reconstructed segments. They used two different datasets for training: 1133 healthy T1-weighted (T1) and 135 healthy contrast-enhanced T1 (T1c) brain MRI scans for detecting AD and brain metastases/various diseases, respectively. Their MADGAN model can detect AD in very early T1 scans and mild cognitive impairment (MCI), with an area under the curve (AUC) of 0.727 and advanced AD with an AUC of 0.894. At the same time, it can detect brain metastases in T1c scans.

To overcome the current problems of deep learning approaches used in diagnosing brain tumors, we still urgently need effective methods to diagnose brain tumors more accurately in their early stages and more quickly to save time for doctors and increase the patient’s survival rate [32,33]. Therefore, the proposed deep wavelet autoencoder (DWAE) novelty is used to solve the problem of low performance, low loss validation, and longtime processing while using MR brain big data analysis. The rest of the paper is organized as follows: Section 1, introduction; Section 2, related work; the dataset collection is given in Section 3; the methodology of the proposed model is given in Section 4; the experimental results and discussion are given in Section 5; the conclusion and future works are given in Section 6.

## 3. Datasets

**Figure 1:**
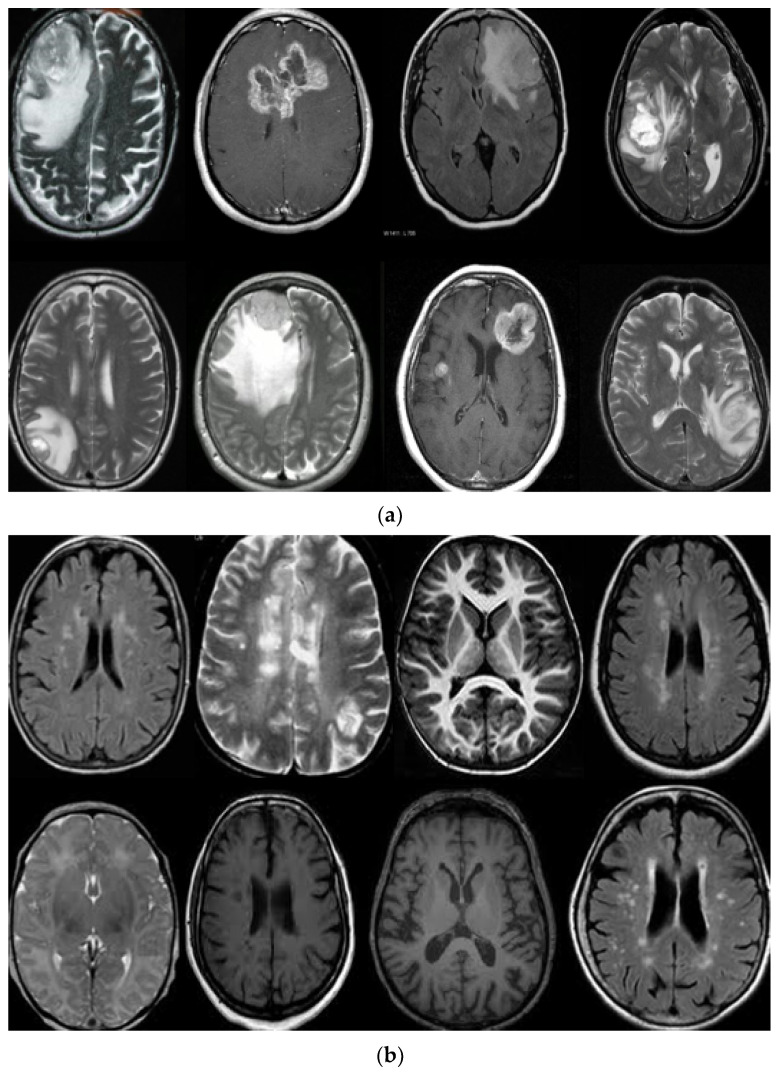
(**a**) describe T1, T2-weighted, and FLAIR MR brain images with tumor and (**b**) describes T1, T2-weighted, and FLAIR MR brain images without the tumor.

In this work, we used five types of MR brain databases, including BRATS2012, BRATS2013, BRATS2014, 2015 challenge, and Brats 2015, and ISLES. Figure 1 shows different types of databases.

## 4. Methodology

**Figure 2:**
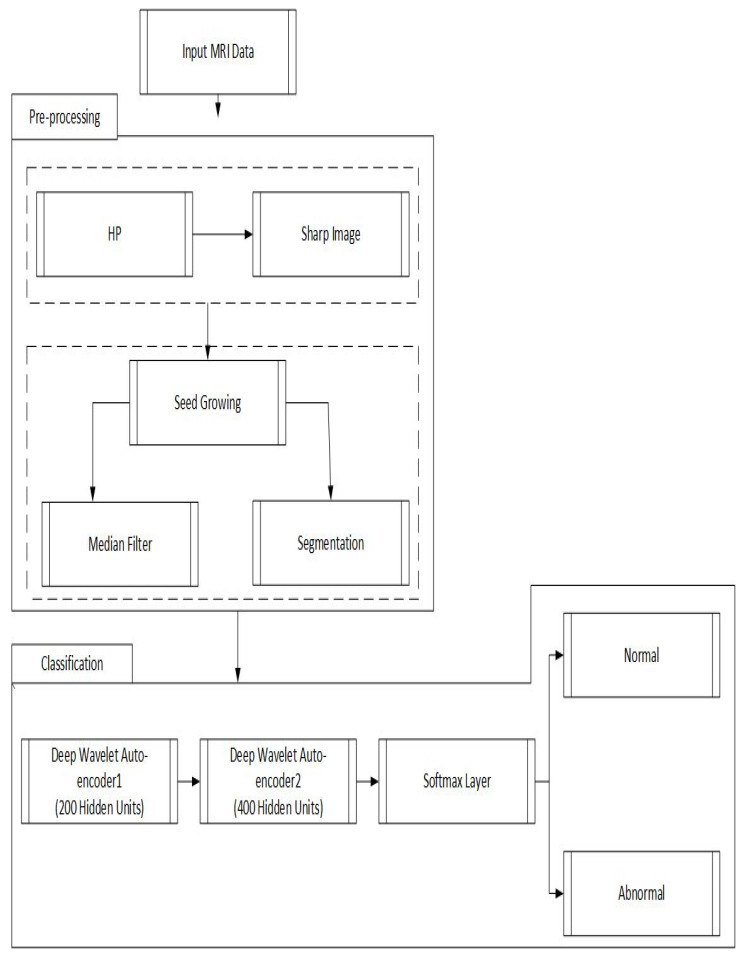
Proposed deep wavelet auto-encoder model architecture steps.

The proposed model is performed in three different stages. In the first stage, a high pass filter [41] and the median filter [42] are selected to enhance input MR brain images. In the second stage, the seed growing approach [43] is utilized to segment the brain tumor. Finally, in the third stage, the segmented images are supplied to the DWAE model. Every two hidden layers are being used, which are further connected with a softmax layer for classification. Figure 2 shows the proposed approach stages.

For more details, the main stages of the proposed approach are as follows:

1. The pre-processing stage is through an enhancement filter, to improve the image; we introduce a new fusion method. In this step, the input MR brain images were resized by 256 × 256 × 1. Then, we choose a high pass filter to improve the edges of the input MR brain image. The input and output of the MR brain image are fused serially. Finally, a combined, fused MR brain image is smoothed using a 3 × 3 median filter that gives the excellent effect of segmentation results compared with previous models.
2. We applied a seed-growing algorithm based on the optimal threshold for good segmentation for a brain tumor.
3. In classification, we applied a deep wavelet auto-encoder (DWAE) model. In this stage, the segmented MR brain image is resized by 256 × 256 × 1 dimension for faster processing. The objective of this stage is to predict the slices with tumor (abnormal MR brain images and the slices without tumor (normal MR brain images).

### 4.1. Deep Wavelet Auto-Encoder

**Figure 3:**
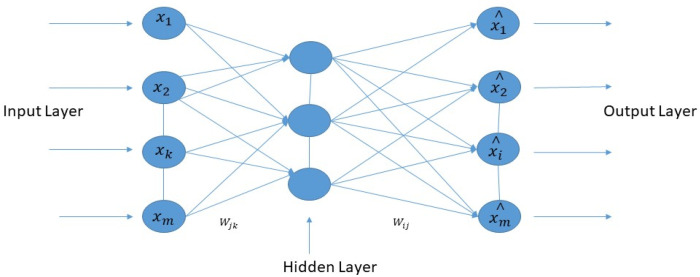
The structure of the wavelet auto-encoder.

**Figure 4:**
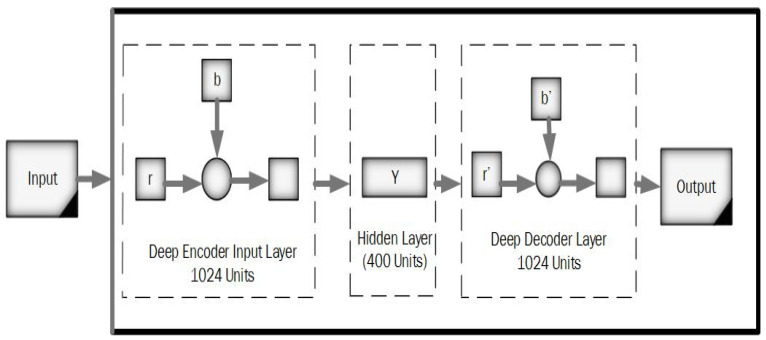
A model of the deep auto-encoder.

The normal auto-encoder features a strong inference ability, robustness, and unsupervised feature learning ability. The property of the Wavelet transform has focal features and time-frequency localization. Therefore, it is essential to combine standard auto-encoder and wavelet transform to solve the practical problems. This article proposed a new kind of improved unsupervised neural network called the “deep wavelet auto-encoder” model, which can catch non-stationary vibration signals and represent complex information. The wavelet auto-encoder model utilized the wavelet function as the activation function in a conventional state, defining different resolutions. The wavelet auto-encoder structure is shown in Figure 3, and the model of the deep auto-encoder is shown in Figure 4.

Figure 4 represents the working principle of the deep auto-encoder, and Equation (1) indicates the decoding stages.

(1) X=ξ(κ^ Y′+b′)

where $\hat{X}$ indicates the outcome of the reconstructed vector, *k* represents kernel vector, $b^{\prime }$ denotes bias value, and ∈ is an error value added during backpropagation.

#### Deep Wavelet Auto-Encoder Model Training

Training samples $y={\left[y_{1},y_{2},\ldots ,y_{n}\right]}^{A}$ the output of the hidden unit is *i*.

(2) $$g_{i}(out)=\varphi \frac{\left(\sum_{i-1}^{n}v_{ij}y_{l}-e_{i}\right)}{b_{i}}$$

where:

*φ* Represents the wavelet activation function.

$y_{l}\left(r=1,2,\ldots ,n\right)$ Is the lth dimension input of the training sample?

$v_{ij}(i=1,2,3,\ldots ,g)$ Is the connection weight between input unit l and the hidden unit *i*.

$b_{i}$ and $e_{i}$ represent $v_{ij}(i=1,2,3,\ldots ,g)$ sent the scale factor and shift factor of wavelet activation function for a hidden unit *i*.

(3) $$\varphi \left(a\right)=\cos\left(5a\right)\exp\left(a^{2}/2\right)$$

(4) $$g_{i}(out)=\varphi_{b.e}\left(i\right)=\cos\left(5\times {\frac{\left(\sum_{l-1}^{n}v_{il}y_{l}-e_{i}\right)}{b_{i}}}^{2}\times {\left(-\frac{1}{2}\frac{\sum_{l-1}^{n}v_{ij}y_{l}-e_{i}}{b_{i}}\right)}^{2}\right)$$

Similar to a normal auto-encoder (AE), we choose the output layer’s activation function as *sigmoid* function. T hen, the output of the deep wavelet auto-encoder can be calculated as in Equation (5):

(5) $$\hat{y}=sigm\left(\sum_{i-1}^{q}v_{ri}\left(\cos5\times \frac{\left(\sum_{i-1}^{n}v_{il}y_{l}-e_{i}\right)}{b_{i}}\times \exp{\left(\frac{-1}{2}\frac{\left(\sum_{l-1}^{n}v_{il}y_{l}-e_{i}\right)}{b_{i}}\right)}^{2}\right)\right)$$

where $\hat{y}$ is *i* the reconstructed dimension output of the training samples, and *v_ri_* is the connection weight between hidden *r* and *i*?

### 4.2. High Pass Filter

Due to the fluctuations of the magnetic field, noise appears in the MR image acquisition process. Therefore, an approach was proposed for enhancing the lesion region and remove the noise. In the proposed method, the high pass filter chooses to distinguish the edges in the input *I*. We obtained the sharper image *sh_I_* by combining *I* with the filtered image *hp_I_* as in Equation (6):

(6) $$sh_{I(x,y)}=I(x,y)+hp_{I(x,y)}$$

To smooth the sharper image’s intensities, we utilized the median filter with a 3 × 3 window size.

### 4.3. Segmentation Using a Seeded Region Growing

By using a seed growing method, brain tumor segmentation is performed. The identical pixel values are grouped when different iterations are completed. We repeated this process until the area stops growing. Equation (7) mathematically describes the predicated function *p_rI_* utilized in the seed-growing algorithm.

(7) $$pr_{I(x,y)}=\{{}_{0}^{1}\;\mathrm{If}\;\mathrm{the}\;\mathrm{difference}\;\mathrm{between}\;\mathrm{the}\;\mathrm{seed}\;\mathrm{point}\;\mathrm{and}\;\mathrm{current}\;\mathrm{pixels}\;\leq \;T\;\mathrm{otherwise}$$

*T* Denotes the predefined threshold.

### 4.4. Softmax Classifier

This paper chooses the softmax to connect the deep wavelet network as follows in Equation (8).

(8) $$\psi \left(Y_{i}\right)=\frac{ek_{i}Y_{i}}{\sum_{i-j}^{N}ek_{j}Y_{i}}$$

where *Y_i_* indicates the output of DWAE.

The softmax classifier *ψ* is utilized for classification based on probability. *k_i_* Denotes *i*th neuron until kernel vector and *N* shows total classes. The output *ψ*(*Y_i_*) shows the *i*th class probability.

The segmented MR brain images are provided for training and testing in the DWAE model. The input image is resized to 32 × 32 × 500, where 500 corresponds to the number of the slices. The input image 256 × 256 describes that 1024 units are provided to the DWAE model. The input with 200 hidden units is passed to DWAE1. DWAE1 with weight initial feature vector and bias is produced, which is given as input to the DWAE2. In DWAE2 400, hidden units are utilized with weight, preference, and hyperparametrs to the produced feature vector. We utilized the softmax layer to perform the classification based on the feature vector acquired from DWAE2.

## 5. Experimental Results and Discussion

In this work, the deep wavelet auto-encoder model is used for training and testing using different databases, as shown in the dataset section. Database 2012 consists of 500 MR brain images in the training stage and 500 MR images, including abnormal and normal in the testing stages [43]. The BRATS2013 database consisting of 1000 MR brain images is used [15]. The BRATS2014 database consists of 800 MR brain Images [44]. The 2015 challenge and Brats 2015, ISLES consists of 700 MR brain images [45]. The input slices consist of abnormal and normal MR brain images from the datasets above. We ran this simulation using a ThinkStation P620 Tower Workstation, NVIDIA Quadro^®^ P2200 16 GB, Lenovo Company, Tianjin City, China. To create a DWAE architecture, we used Tensorflow and Keras, Spyder 3.6. We tested different databases to evaluate and analyze different factors in the deep wavelet auto-encoder model. The results of the experiments include two steps. Firstly, the results of MR database processing, and secondly, performance metrics.

### 5.1. The Results of MR Database Processing

**Figure 5:**
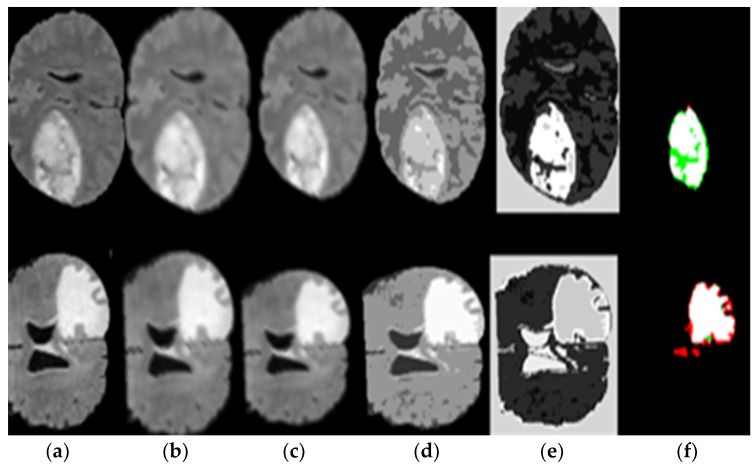
(**a**) Original image, (**b**) image sharpening, (**c**) high pass filter, (**d**) seed growing, (**e**) applied thresholding, and (**f**) segmented tumor alone.

**Figure 6:**
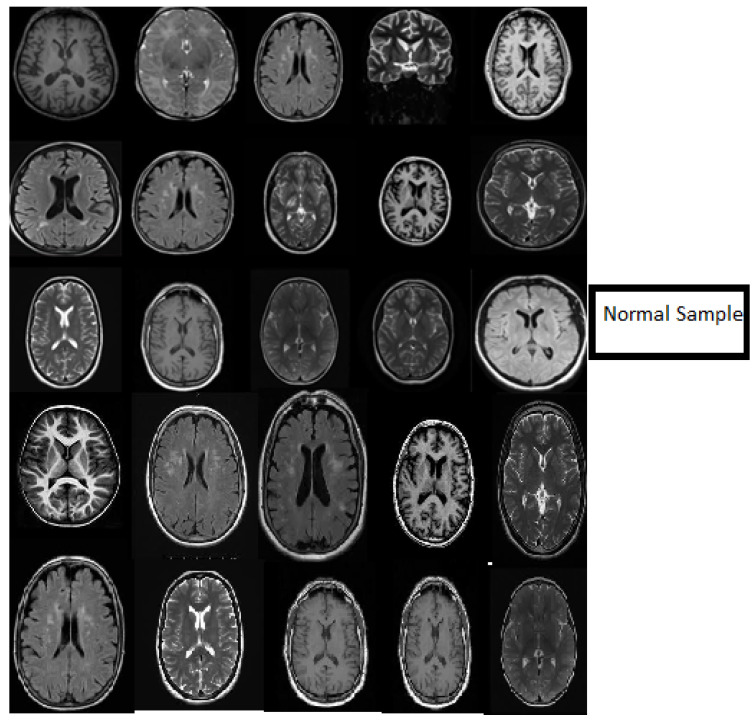
Samples showing the results of MR brain normal images classification using the deep wavelet auto-encoder model.

**Figure 7:**
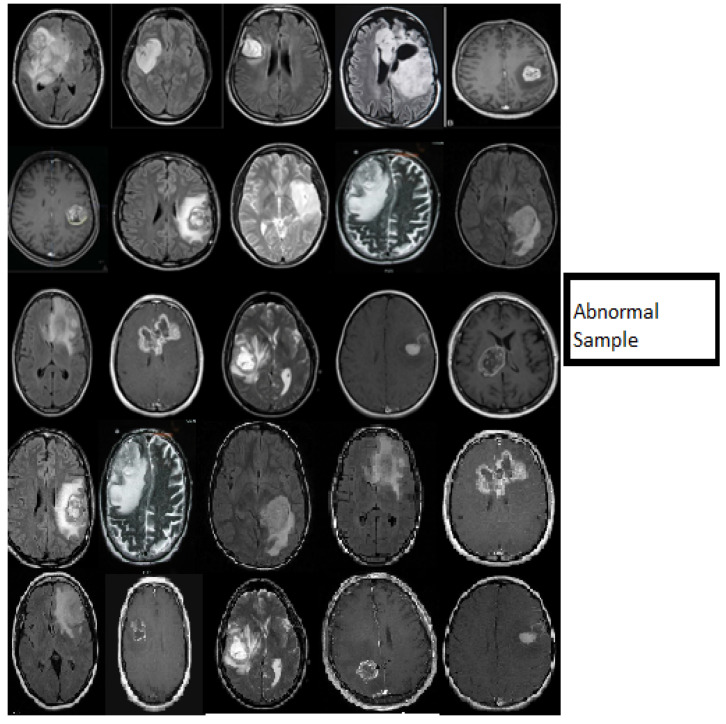
Samples showing the results of MR brain abnormal images classification using the deep wavelet auto-encoder model.

This section used the deep wavelet auto-encoder model to process MR dataset and the segmentation method described in Figure 5 and the classification method, as shown in Figure 6 and Figure 7.

Figure 5 represents the results of pre-processing steps based on the segmentation method, which consists of the (a) original image, (b) the result of applied image sharpening, (c) the results of applied high pass filter, (d) the result of applied seed growing, (e) the result of applied thresholding and (f) the result of the applied segment tumor area. Figure 5 proves the excellent ability of the proposed DWAE model during pre-processing stage and segmentation.

Figure 6 describes the classification stage and shows the excellent capacity of the proposed model in the classification stage using normal MR databases. The slice consists of 15 MR normal brain images processed using a classification method based on the DWAE model, and the output shows a good result. Figure 7 represents the classification stage, and notable is the perfect ability of the proposed model in the classification stage using an abnormal database. The slice consists of 15 MR abnormal brain images processed using the classification method based on the proposed model. The results show the robustness of the model to classify the brain tumor. Figure 6 and Figure 7 prove the ability of the proposed model to predict the normal MR brain images and abnormal MR brain images. Generally, the model demonstrated a high ability to process, detect, and classify brain tumors using MR images.

**Figure 8:**
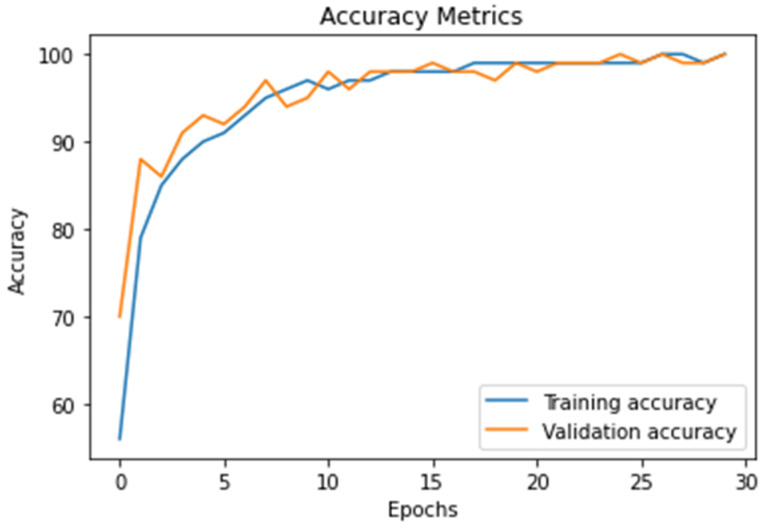
The result of the proposed model accuracy value and the model’s accurate and robust model during training and testing stages as calculated using Equation (10).

The Figure 8 represents the accuracy value of DWAE model (training and testing), the graph shows the effectiveness of the proposal model by achieved accuracy of 99.3%.

The loss value (training and testing) means that validation loss has the same metric as training loss, but it is not utilized to update the weights. It is calculated similarly by running the network forward over inputs *x_i_* and comparing the network outputs $\hat{y_{i}}$ with the ground truth-values using a loss function (9).

(9) $$J=\frac{1}{N}\sum_{i-1}^{N}\zeta \left(\hat{y_{i}},y_{i}\right)$$

**Figure 9:**
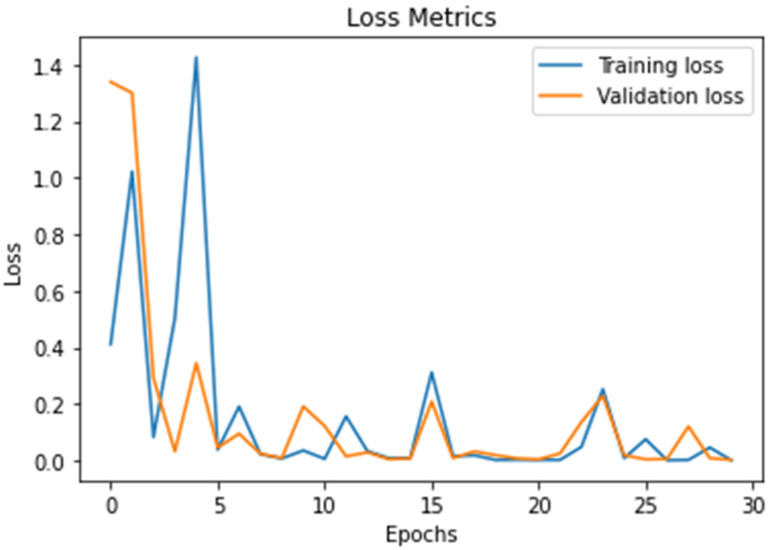
Describe the results of the loss value using the DWAE model.

Figure 9 presents the proposed DWAE model loss value; the results show that the proposed model achieved (0.1 to 0.3) a best loss validation value during the training and testing stages. We trained and tested the proposed model using 30 epochs during the accuracy and loss validation stages.

### 5.2. Performances Metrics

To evaluate the proposed DWAE model efficiency, we compare the proposed model overall performance using accuracy, sensitivity, specificity, DSC, Precision, JSI, FPR, and FNR values.

#### 5.2.1. Accuracy (ACC)

Accuracy (ACC) is utilized to compute the degree of correct tumor classification rate, and is calculated using the following Equation (10):

(10) $$\mathrm{Accuracy}=\frac{\left(\mathrm{TP}+\mathrm{TN}\right)}{\left(\mathrm{TP}+\mathrm{TN}\right)+(\mathrm{FP}+\mathrm{FN})}\times 100$$

#### 5.2.2. Sensitivity (SE)

Sensitivity (SE) is utilized to calculate the degree of how much approach is sensitive to measure the tumor identification rate, and is calculated using the following Equation (11):

(11) $$\mathrm{Sensitivity}=\frac{\left(\mathrm{TP}\right)}{\left(\mathrm{TP}+\mathrm{FN}\right)}\times 100$$

#### 5.2.3. Specificity (SP)

Specificity (SP) is the rate between true negative (TN) and true positive (TP), and is calculated using the following Equation (12):

(12) $$\mathrm{Specificity}=\frac{\left(\mathrm{TN}\right)}{\left(\mathrm{TN}+\mathrm{FP}\right)}\times 100$$

#### 5.2.4. Dice Similarity Coefficient (DSC)

Dice similarity coefficient (DSC) is utilized to compute the ratio between the actual tumor and non-tumor, which are compared with predicted tumor and non-tumor pixels, and is calculated using the following Equation (13):

(13) $$\mathrm{DSC}=\frac{2\mathrm{TP}}{\mathrm{FP}+2\mathrm{TP}+\mathrm{FN}}\times 100$$

#### 5.2.5. PRECISION (PRE)

PRECISION (PRE) describes the number of digits that are used to express a value, and is calculated using the following Equation (14):

(14) $$\mathrm{Precision}=\frac{\left(\mathrm{TP}\right)}{\left(\mathrm{TP}+\mathrm{FP}\right)}\times 100$$

#### 5.2.6. JACCARD Similarity Index (JSI)

JACCARD similarity index (JSI) is utilized to compute the similarity between the actual tumor pixels and predicted tumor pixels and is calculated using the following Equation (15):

(15) $$\mathrm{JSI}=\frac{\mathrm{TP}}{\mathrm{TP}+\mathrm{FN}+\mathrm{FP}}\times 100$$

#### 5.2.7. FALSE Positive Rate (FPR)

FALSE positive rate (FPR) is utilized to compute the ratio of wrongly identified pixels, corrected identified pixels, and is calculated using the following Equation (16):

(16) FPR=1−Specificity

#### 5.2.8. FALSE Negative Rate (FNR)

FALSE negative rate (FNR) is utilized to compute the positive proportion, but the approach-identified negative and is calculated using the following Equation (17).

(17) FNR=1−Sensitivity

where true positive (TP), true negative (TN), false positive (FP), and false negative (FN).

The proposed DWAE model is evaluated by comparing 21 existing models on machine learning methods based on the accuracy (ACC), sensitivity (SE), specificity (SP), dice similarity coefficient (DSC), precision (PR), Jaccard similarity index (JSI), false-positive rate (FPR), and false-negative rate (FNR) values as shown in the Table 1, Table 2, Table 3 and Table 4.

Table 1 compared the proposed DWAE model results with 10 previous models published in the best level indexed. Deep wavelet autoencoder with deep neural network DWA-DNN model [29] for brain MRI image classification for cancer identification achieved an accuracy of 93.14%, sensitivity of 92.16%, and specificity of 94.26%, and precision of 94.81%.

Brain tumor detection using the convolutional neural network (CNN) model [46] obtained an accuracy of 96.5%, specificity of 95%, and a precision of 89.66%. VGG16 model for brain classification and analysis [47] achieved an accuracy of 84.48%, sensitivity of 81.25%, specificity of 88.48%, and precision of 89.66%.

Brain tumor classification using the k-nearest neighbors (KNN) model [48] obtained an accuracy of 78%, a sensitivity of 46%, and a specificity of 50%. The deep neural network (DNN) model for brain cancer detection [49] achieved an accuracy of 93%, sensitivity of 75%, specificity of 80%, and precision of 72%.

Brain tumor classification using modified convolutional neural network (M-CNN) model [50] obtained an accuracy of 96.4%, a sensitivity of 95%, a specificity of 93%, and a precision of 95.7%. Brain tumor segmentation using deep auto-encoder with Jaya optimization algorithm (DAE-JOA) model [33] achieved an accuracy of 98.5%, sensitivity of 95.4%, and precision of 95.6%.

A hybrid convolutional neural network with a self-vector machine (CNN-SVM) model [51] for brain tumor classification obtained an accuracy of 95.62%, specificity of 95%, and precision of 92.12%. Brain tumor identification using the Alex-Net model [52] achieved an accuracy of 87.66%, sensitivity of 84.38%, and specificity of 92.31%. Google-Net model [47] for brain tumor detection and analysis obtained an accuracy of 89.66%, a sensitivity of 84.85, specificity of 96%, and precision of 97.4%. The deep features model [53] for brain tumor achieved an accuracy of 98.71% and specificity of 96.71%. U-NET model [54] for brain tumor detection obtained an accuracy of 98.72% and sensitivity of 90.7%. The proposed deep wavelet auto-encoder (DWAE) model obtained an accuracy of 99.3%, sensitivity of 95.6%, specificity of 96.9% and precision of 97.4%.

Based on the results of 10 existing models shown in Table 1, we conclude that the proposed deep wavelet auto-encoder (DWAE) model achieved better performance than previous models using accuracy, sensitivity, specificity, and precision values.

Table 2 describes a comparison between the proposed deep wavelet auto-encoder (DWAE) models results with existing models are based on dice similarity coefficient (DSC) value achievement. Brain tumor classification using the convolutional neural network (CNN) model [20] achieved a DSC of 83.7%. The convolutional neural network small filter model [49] obtained a DSC of 88% for brain tumor classification. Brain tumor detection using the conditional random fields (CRF) model achieved a DSC of 62%.

**Table 2:** Comparison of the existing model with the proposed DWAE model using DSC value.

Model	DSC%
CNN [20]	83.7
CNN-small filter [49]	88
CRF [55]	62
HMV [45]	85
3D fully connected [21]	84.7
Integrated hierarchical [56]	73
Local independent projection [57]	75
RG + MKM + U-NET [54]	90
HOG + LBP + deep features [53]	96.11
Multi-scale 3D with fully connected CRF [21]	90
Proposed DWAE model	96.55

The hierarchical majority vote (HMV) model for brain tumor detection [45] obtained a DSC of 85%. Brain tumor classification using a 3D fully connected model achieved a DSC of 84.7%. The integrated hierarchical model [47] for brain tumor detection obtained a DSC of 73%.

Local independent projection model [57] for brain tumor classification achieved a DSC of 75%. Brain tumor classification using multi-scale 3D with fully connected CRF model [21] achieved DSC of 90%. The deep features model [53] for brain tumor achieved a DSC of 90%. U-NET model [54] for brain tumor detection obtained a DSC of 96.1%. The proposed DWAE model obtained a DSC of 96.55%.

According to the results shown in Table 2, based on the nine previous models, we noticed that the proposed deep wavelet auto-encoder (DWAE) model achieved a better dice similarity coefficient (DSC) value than the existing models.

Table 3 presented the comparison results of the proposed DWAE model with existing models using FPR and FNR values. Brain tumor detection using the deep neural network (DNN) model [58] achieved FPR 0.16 and FNR 0.06. The deep autoencoder with Jaya optimization algorithm (DAE-JOA) model for brain tumor detection [33] obtained FPR 0.46 and FNR 0.04. Stacked auto-encoder model for brain tumor identification [32] achieved FPR 0.07 and FNR 0.1. Brain tumor classification based on the Alex-Net model [52] obtained FPR 0.07 and FNR 0.339. Google-Net model [47] for brain tumor detection achieved FPR 0.714 and FNR 0.339. Brain tumor identification using k-nearest neighbors (KNN) model [48] obtained FPR 0.62 and FNR 0.54. The multimodal model for brain tumor classification using deep learning model [59] achieved an FNR of 1.74. The proposed DWAE model achieved FPR 0.0625 and FNR 0.031. Based on the results shown in Table 3, the proposed model produced better results using FPR and FNR values than the previous models.

**Table 3:** Comparison of the existing models with the proposed DWAE model using FPR and FNR values.

Model	FPR	FNR
DNN [58]	0.16	0.06
DAE-JOA [33]	0.46	0.04
Stacked auto-encoder [32]	0.07	0.1
Alex-Net [52]	0.07	0.128
Google-Net [47]	0.714	0.339
Multimodal [59]	-	1.74
KNN [48]	0.62	0.54
Proposed DWAE model	0.0625	0.031

Table 4 describes a comparison of the proposed DWAE model’s results with an existing model based on the JSI value. The hybrid stacked auto-encoder based on deep learning model [32] for brain tumor detection obtained JSI of 89%. A deep neural network model [58] for MR big data analysis achieved a JSI of 90.4%. A stable algorithm based on a deep learning model [28] for automated segmentation using MR FLAIR images obtained a JSI of 92.3%. Our proposed model achieved a JSI of 93.3%.

**Table 4:** Comparison of the previous model with the proposed DWAE model using JSI value.

Model	JSI%
Stacked auto-encoder [32]	89
DNN [52]	90.4
Stable algorithm [28]	92.3
Proposed DWAE model	93.3

The proposed DWAE model is compared with 21 previous models such as [20,21,28,29,32,33,45,46,47,48,49,50,51,52,53,54,55,56,57,58] and [59], as shown in Table 1, Table 2, Table 3 and Table 4 based on accuracy, sensitivity, specificity, precision, DSC, FPR, FNR, and JSI values. According to the performance and analysis of the previous models above, the results suggest that the proposed model performed very well compared to previous models.

In this work, we discuss threats-to-validity of the experimental results using 21 existing models published in the top-level journals in the field of brain tumor detection and classification using the same databases as shown in Table 1, Table 2, Table 3 and Table 4. The validation of our results is based on a comparison between existing models with the proposed deep wavelet auto-encoder model using eight values. The ability of the proposed model to be validated on other BRATS big databases with high accuracy and very low loss validation, as shown in Figure 8 and Figure 9, show a clear evidence of the threat-validity of the results.

## 6. Conclusions

Deep learning networks models have obtained good results in recent years in the medical image analysis field. In this model, we implemented the necessary phases such as image sharpening, high pass filter, thresholding segmentation, growing seed approach, and classification based on deep wavelet auto-encoder model for feature extraction; the implementation produced excellent results, as shown in the results experiments in Figure 5, Figure 6 and Figure 7.

The proposed model testing and training combine databases from BRATS2012, BRATS2013, BRATS2014, 2015 challenge, and Brats 2015. The average accuracy is 99.3%, sensitivity 95.6%, specificity 96.9%, precision 97.4%, DSC 96.55%, FPR 0.0625, FNR 0.031, and JSI 93.3%, respectively. Based on the overall experiment’s output, segmentation, classification, and performance of the proposed DWAE model, we conclude that the proposed model achieved better results than the 21 existing models published in high-level journals.

The advantage of this proposed method is its excellent ability to analyze large data from magnetic resonance images of the brain without technical problems and with very high accuracy, which will help doctors in the accurate diagnosis of brain tumors.

Our proposed model achieved a great overall performance on brain tumor identification and classification stages, allowing the model to be used in computing techniques for the early detection of brain tumor. DWAE model shows the importance of the deep learning model in the medical field and medical applications. In future work, we will evaluate the overall performance of our DWAE model. We will improve the different layer parameters that are hidden in the model to increase the accuracy and make the model faster. We will validate our proposed model using the BRATS 2019 and BRATS 2020 datasets.

## Figures and Tables

**Table 1:** Comparison of the results of previous models with the proposed DWAE model.

Model	Accuracy %	Sensitivity %	Specificity %	Precision %
DWA-DNN [29]	93.14	92.16	94.26	93.15
DAE-JOA [33]	98.5	95.4	-	95.6
CNN [46]	96.5	-	95	94.81
Google-Net [47]	89.66	84.85	96	96.55
Vgg16 [47]	84.48	81.25	88.48	89.66
KNN [48]	78	46	50	52
DNN [49]	93	75	80	72
M-CNN [50]	96.4	95	93	95.7
CNN-SVM [51]	95.62	-	95	92.12
Alex-Net [52]	87.66	84.38	92.31	93.1
HOG + LBP + Deep features [53]	98.71	98.46	96.72	-
RG + MKM + U-NET [54]	98.72	90.7	99.7	-
Proposed DWAE Model	99.3	95.6	96.9	97.4

## Data Availability

The data presented in this study are available on request from the corresponding author.
